# Circularly Polarized Light Detection by Chiral Photonic Cellulose Nanocrystal with ZnO Photoconductive Layer in Ultraviolet Region

**DOI:** 10.3390/nano11113098

**Published:** 2021-11-16

**Authors:** Boyu Zhang, Sixiang Zhao, Yingying Yu, Ming Li, Liancheng Zhao, Liming Gao

**Affiliations:** State Key Laboratory of Metal Matrix Composites, School of Material Science and Engineering, Shanghai Jiao Tong University, Shanghai 200240, China; zhboyu123@sjtu.edu.cn (B.Z.); zhaosixiang@sjtu.edu.cn (S.Z.); yuyingying@sjtu.edu.cn (Y.Y.); mingli90@sjtu.edu.cn (M.L.); lczhao@sjtu.edu.cn (L.Z.)

**Keywords:** circularly polarization light detection, cellulose nanocrystal, ZnO photoconduction

## Abstract

Circularly polarized light (CPL) detection and polarization state recognition are required for a wide range of applications. Conventional polarization detection with optical components causes difficulties for miniaturization and integration. An effective design strategy is proposed for direct CPL detection with chiral material. Here, we realized direct CPL detection based on the combination of chiral photonic cellulose nanocrystal (CNC) and ultraviolet-sensitive ZnO photoconductive material. The CNC layer deposited by evaporation-induced self-assembly established the left-handed chiral nematic structure with a photonic bandgap (PBG) to recognize left-handed CPL (LCPL) and right-handed CPL (RCPL) at specific wavelengths. The PBG of CNC layer has been modulated by the adjustment of chiral nematic pitch to match the semiconductor bandgap of ZnO film in ultraviolet region. The photocurrents under RCPL and LCPL are 2.23 × 10^−6^ A and 1.77 × 10^−6^ A respectively and the anisotropy factor Δ*g_pc_* of 0.23 is acquired for the CPL detection based on the chiral photonic CNC. This design provides a new approach to the detection of CPL polarization state with competitive performance.

## 1. Introduction

Circularly polarized light (CPL) has attracted great interest in a wide range of applications, from optical communication [1] and quantum computing [2,3,4] to biosensor [5] and substance screening [6]. Conventional polarization detection requires the assistance of optical components by integrating a non-chiral photodetector with a quarter-wave plate and a linear polarizer [7,8], but it is difficult to realize miniaturization and integration. Unlike indirect detection which requires optical elements, direct detection of CPL by chiral materials with intrinsic advantages can be exploited for integration in more applications [9]. Directly detectable electronic circuits for CPL can be created by a heterojunction photodiode [10,11,12], field-effect transistor [9], and plasmon resonance [13] to distinguish between different polarization states of CPL. Chiral organic semiconductors can be integrated as the photoactive layer in bulk heterojunction photodiodes to convert CPL into a polarization-dependent photocurrent [10,14]. Chiral hybrid organic-inorganic perovskites induce chirality into inorganic sublattice band edge states for efficient charge transport [11]. Chiral metamaterials based on plasmonic elements generate photocurrent from hot carrier generation and injection [13]. Seeking suitable materials with strong chirality is a main challenge to direct CPL detection [15], and chiral cellulose nanocrystals (CNCs) offer an appealing opportunity for integrated CPL detector.

CNCs are a kind of chiral material that is abundant in nature and easy to extract. CNCs are highly crystalline rod-like nanorods (diameter 3–20 nm) that can be isolated by sulfuric acid hydrolysis. After surface-functionalizing with −OSO^3−^ groups, the nanorods are negatively charged. The electrostatic repulsion between the CNCs results in the formation of stable suspensions [16]. When the CNC suspensions reach a certain critical concentration, they self-assemble into a left-handed chiral nematic liquid crystal [17,18]. The chiral nematic structure can be preserved from the CNC suspensions in the resultant film when evaporated under controlled conditions [19]. The CNC film is a one-dimensional photonic crystal with strong optical anisotropy [20,21]. It selectively reflects left-handed circularly polarized light (LCPL) and transmits right-handed circularly polarized light (RCPL) in its photonic bandgap (PBG) [22,23]. The PBG of CNC photonic crystal is mainly determined by Bragg’s law:

(1) $$\lambda =n_{av}p\cos\theta ,$$

where *n_av_* is the average refractivity of the extraordinary and ordinary optical indices in the phase, while *p* is chiral nematic pitch and *θ* is the incident angle of light with respect to the helical axis of chiral nematic phase in CNC film. The PBG of CNC film is intrinsically related to the pitch, which is influenced by the initial CNC suspension and its drying process. For instance, sonication treatment with increasing energy input destroys the CNC aggregates in suspension, resulting in pitch increase and red-shift of the PBG in CNC film. On the contrary, addition of electrolyte strongly decreases the pitch in CNC suspensions by decreasing the strength of the electrostatic repulsion between nanorods [24,25]. Several mechanical and chemical methods can change the chiral nematic pitch of CNC films to conveniently modulate the PBG [26], exhibiting great potential as chiral material for CPL detection.

The strong optical anisotropy based on CNC chiral material should translate into photocurrent anisotropy by the reasonable device design. Photoconductive materials as photosensitive substrate in CPL detector attract attention for their easy fabrication and fast response. ZnO is a typical photoconductive material with wide bandgap (3.37 eV) which has a wide range of applications, e.g., solar cell [27], optoelectronic devices [28], and ultraviolet (UV) photodetector [29], due to the advantages of safety, high responsivities, and obvious visible blindness [30,31,32,33,34]. ZnO layer can be prepared by magnetron sputtering, sol-gel, spray pyrolysis [35,36,37]. The sol-gel method is chosen for simple equipment, low cost and large coating area. Since ZnO shows promising prospects of UV photodetection, the integration of CNC chiral photonic material with ZnO photoconductive detector has great potential for CPL detection in the UV region.

In this work, we propose a new optoelectronic device to realize the direct detection of circular polarization states with the combination of chiral photonic CNC material and ultraviolet-sensitive photoconductive ZnO material. The CNC layer was deposited on a ZnO layer by evaporation-induced self-assembly (EISA). The PBG of CNC layer has been modulated to match the bandgap of ZnO photosensitive semiconductor to maximally convert the selective reflection of CPL into an electrical signal. The strong optical chirality of CNC ensures high performance to distinguish between LCPL and RCPL without conventional optical components. The resulting CPL-UV detector reports to have the photocurrent anisotropy factor Δ*g_pc_* of 0.23 in the UV region, realizing high performance CPL photodetection.

## 2. Materials and Methods

### 2.1. Materials

Microcrystalline cellulose (MCC, 11 wt%) was purchased from Sigma-Aldrich Chemistry (St. Louis, MO, USA). Zinc acetate dihydrate (Zn(Ac)_2_·2H_2_O, 99%) and monoethanolamine (MEA, 99%) were purchased from Aladdin (Shanghai, China). Sulfuric acid (H_2_SO_4_, 95%) and 2-methoxyethanol (C_3_H_8_O_2_, 99.5%) were purchased from Sinopharm Chemical Reagent Co. (Shanghai, China). All the chemicals were directly used without further purification.

### 2.2. Preparation of the Cellulose Nanocrystal Suspension

The cellulose nanocrystal was prepared from MCC. 10 g MCC was added to 100 mL 64% sulfuric acid mixed by 50 mL de-ionized water and 50 mL 98% concentrated sulfuric acid in ice bath. The suspension was kept in hot water bath at 45 °C for 1 h with vigorous stirring. The suspension was then diluted with cold de-ionized water (~10 times the volume of the acid solution used) to stop the hydrolysis and settled overnight. The clear top layer was removed and the remained precipitation was washed by centrifugation with de-ionized water for several times to remove acidic solution. The sediment was collected in dialysis membrane tubes and dialyzed against de-ionized water. The dialysis process lasted for two weeks. After that, the suspension was subjected to sonication treatment for 5 min in an ice bath to disperse uniformly.

### 2.3. Preparation of the ZnO Layer

The ZnO layer was prepared with spin coating by the sol-gel method. Thus, 0.8 M Zn(Ac)_2_·2H_2_O was first dissolved in 100 mL 2-methoxyethanol. MEA was then dropped into the solution as stabilizer. The molar ratio of MEA and Zn(Ac)_2_·2H_2_O was kept at 1:1. The solution was kept vigorous stirring in hot water bath at 60 °C for 1 h to form a transport sol. After that, the sol aged for 24 h in dark. The quartz substrate (3 cm × 3 cm) was ultrasonically cleaned with detergent, pure water, and alcohol in turn for 15 min before spin coating. The sol was dropped on the quartz substrate followed by rotating at the spinning speed of 3000 rpm for 30 s. The deposited ZnO layer was then preheated on hot plate at 200 °C for 10 min. The spin coating and preheating procedure was repeated for eight cycles. The deposited ZnO layer was then annealed in ambient air at 600 °C for 1 h.

### 2.4. Device Fabrication

Briefly, 1 mL of CNC suspension was deposited on the ZnO layer with mask on both sides, followed by evaporation at 30 °C to produce the CNC/ZnO photodetector device. The silver (Ag) electrodes with 2 cm spacing were printed on two sides of ZnO layer acted as metal contacts.

### 2.5. Characterization

The cross-sectional morphology of the detector was observed by scanning electron microscopy (TESCAN MIRA, Brno–Kohoutovice, Czech Republic). The height profile of the CNC layers after EISA was measured by a surface profilometer (KLA Alpha-step D600, Milpitas, CA, USA). The crystallization of ZnO layer was determined by X-ray diffractometry (Bruker D8 Advance, Berlin, Germany) with Kα radiation (*λ* = 1.5406 Å) of Cu. Polarized optical microscopy (POM) was performed using a metallurgical microscope (ZEISS Primotech, Oberkochen, Germany). The transmittance spectra and circular dichroism (CD) spectra were collected by UV-visible-NIR spectrophotometry (PerkinElmer Lambda 950, Waltham, MA, USA) and CD spectrometry (JASCO J-1500, Tokyo, Japan). The photoluminescence (PL) spectra were recorded with a photoluminescence spectrometer (Edinburgh FLS1000, Livingston, UK). The photocurrent measurements of photodetector were recorded with an electrochemical workstation (Chenhua CHI760E, Shanghai, China).

### 2.6. Circularly Polarization State Detection Test

The light was generated from light-emitting diode (LED) with a wavelength of 365 nm (Thorlabs M365L2, Newton, MA, USA). The light intensity is approximately 8.9 μW/mm^2^. A linear polarizer (Thorlabs LPUV100, Newton, MA, USA) and a quarter-wave plate (Thorlabs AQWP05M-340, Newton, MA, USA) were used to obtain LCPL and RCPL. The unpolarized light went through the combination of a linear polarizer and a quarter-wave plate with angle *φ* between the polarization direction of the linear polarizer and the fast axis of the quarter-wave plate. The polarization state of the incident light varied with angle *φ*. LCPL and RCPL could be generated respectively when the angle *φ* is −45° and +45°. The current-voltage and current-time curves of CNC/ZnO photodetector under LCPL and RCPL illumination were measured using an electrochemical workstation.

## 3. Results and Discussion

### 3.1. Fabrication and Structure of CNC/ZnO Photodetector

**Figure 1:**
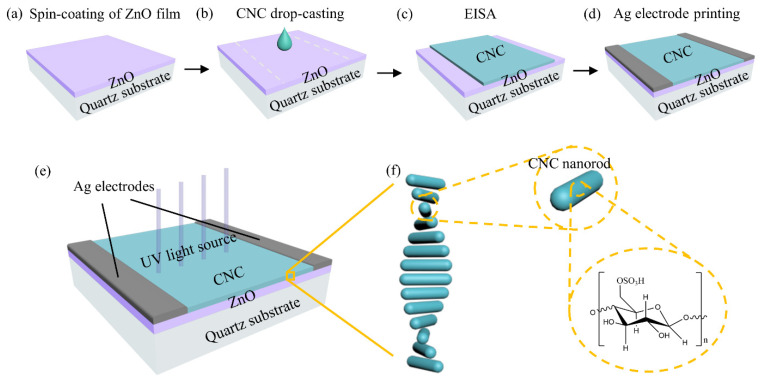
(**a**–**d**) Fabrication flow of the CNC/ZnO CPL photodetector. (**e**) Schematic of CNC/ZnO CPL photodetector. (**f**) Chiral nematic ordering of CNC layer.

Figure 1a–d depicts the detector fabrication flow step by step. In order to fabricate the detector with ZnO as photoconductive UV-sensitive layer, the sol-gel method followed by annealing at 600 °C was used to form ZnO layer on the quartz substrate. Hence, 4.8 wt% CNC suspension from sulfate acid hydrolysis was sonicated with increasing treatment time to modulate the chiral nematic pitch of the CNC film cast from EISA. The peak reflection wavelength of the CNC film increased from 370 nm at 5 min sonication to 650 nm at 20 min sonication (Figure S1). Sonication treatment of CNC suspension for 5 min was selected to modulate the peak reflection wavelength to the UV region. Hence, 1 mL of CNC suspension was drop-casted onto the ZnO substrate and kept at 30 °C for 1 d for EISA. Finally, after CNC layer formation, two Ag contacts as electrodes were printed on two sides of the ZnO layer with constant distance of 2 cm. Figure 1e shows the complete structure of CNC/ZnO CPL photodetector. The photograph of the detector was taken from the normal direction (Figure S2). The CNC layer shows an obvious structure color. A weak coffee-ring effect can be observed at the outer edge of the CNC layer after EISA [38]. The height profile of the CNC layer (Figure S3) also shows ring-shaped deposition due to the higher evaporation rate near the edge. The coffee-ring effect of the CNC/ZnO photodetector is not obvious [39], so it was ignored during the CPL photodetection. Figure 1f provides an insight into the chiral nematic ordering of the CNC photonic layer by EISA.

### 3.2. Morphological and Chiroptical Study

**Figure 2:**
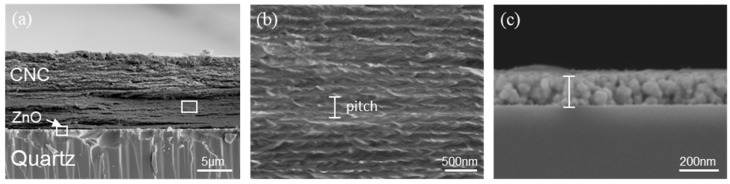
SEM of a cross-section of (**a**) CNC/ZnO photodetector, (**b**) CNC layer and (**c**) ZnO layer.

**Figure 3:**
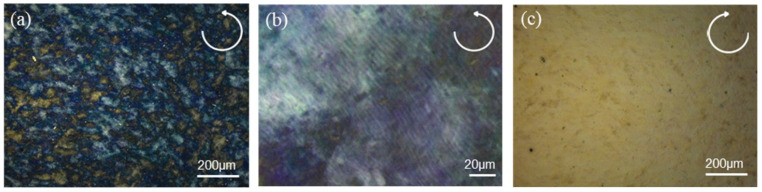
(**a**) POM of CNC/ZnO photodetector under the LCPL reflection mode. The characteristic finger texture in shown in (**b**). (**c**) POM of CNC/ZnO photodetector under the RCPL reflection mode.

Figure 2a shows a scanning electron microscopy (SEM) image of the cross section of the CNC/ZnO CPL photodetector, which are the quartz substrate, ZnO layer and CNC layer from bottom to top. The macroscopic thickness of CNC layer is measured to be 11.12 μm. Figure 2b is a high magnification image of the cross section of the CNC layer, which was cast from the CNC suspension with 5 min sonication treatment. The image shows a periodic layered structure. CNC nanorods are arranged in a regular way to form a periodic chiral nematic arrangement in the CNC layer. The periodic band is a half chiral nematic pitch ($\frac{p}{2}$) which is related to 180° rotation of the chiral nematic direction. The pitch distance of CNC layer with 5 min sonication treatment is measured to approximately 230 nm [40]. Sonication treatment is an effective physical method to modulate the chiral nematic pitch of the CNC layer. Sonication treatment will release the ions in the hydrated layer into the solution, which enhances the dielectronic layer structure of cellulose nanorods. The electrostatic repulsion between the nanorods increases, and consequently increases the pitch, so the peak of reflected wavelength is red-shifted after EISA [41]. The sonication time of 5 min is selected to obtain a CNC layer with peak reflection wavelength in the UV region. The SEM cross section morphology of ZnO layer is shown in Figure 2c. The highly faceted granular ZnO grains stack to form ZnO layer with thickness of 180 nm. The diffraction peaks at 2*θ* = 31.9°, 34.6°, and 36.4° in X-ray diffraction pattern representing (100), (002), (101) planes, exhibit the polycrystalline hexagonal wurtzite structure of ZnO (Figure S4) [29]. Figure 3a–c are POM images of CNC/ZnO photodetector under left-handed and right-handed circularly polarizing filters respectively. The clear blue reflection under the LCPL mode in Figure 3a is observed to completely vanish under the RCPL mode in Figure 3c. In the POM image (Figure 3b), the CNC layer exhibits a fingerprint texture, which is characteristic of chiral nematic phase [25].

**Figure 4:**
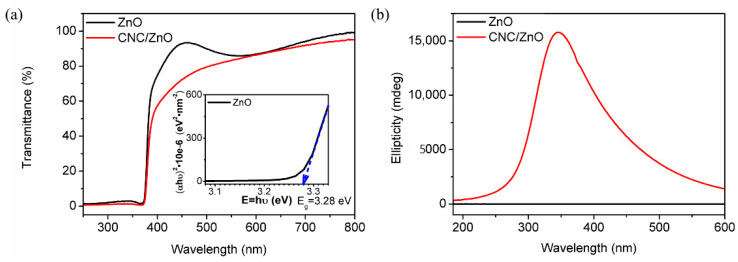
(**a**) Transmittance spectra of ZnO layer and CNC/ZnO photodetector. Insert: Plot of ${\left(\alpha h\nu \right)}^{2}$ versus photon energy for ZnO layer. (**b**) CD spectra of ZnO layer and CNC/ZnO photodetector.

After annealing of the ZnO layer, the CNC suspension was deposited on the surface of ZnO to form the chiral photonic CNC layer. Figure 4a shows the transmittance spectra of the detector between 250 nm and 800 nm. The ZnO layer has a sharp transmittance decrease at the absorption edge of 370 nm in the UV region, which is attributed to the intrinsic ZnO bandgap. The electron transitions from the valence band to the conduction band when ZnO is illuminated by UV light with higher energy than bandgap. The optical bandgap can be obtained using the Tauc model:

(2) $${\left(\alpha h\nu \right)}^{2}=A\left(h\nu -E_{g}\right),$$

where *α* is absorption coefficient, *hν* is photon eneygy, *A* is a constant, and *E_g_* is the optical energy gap. The transmittance and reflectance spectra of ZnO layer is shown in Figure S5. The low reflectance of ZnO layer can be ignored when the absorption coefficient is calculated [42]. The absorption coefficient *α* follows the relation:

(3) $$\alpha =-\frac{1}{d}\ln T\left(\lambda \right)$$

where *d* is the thickness and *T* is optical transmittance. *E_g_* of ZnO layer is measured to be 3.28 eV from Tauc’s plot, which is consistent with the absorption edge of 370 nm. The PL spectra of ZnO layer are measured at room temperature (Figure S6). The PL spectra of ZnO layer has two emission bands. One is in the UV region, which is attributed to the near-band-edge emission through exciton–exciton collision processes. The other is in the visible region, and probably comes from the electron–hole recombination at a deep level emission in the band gap caused by intrinsic point defects and surface defects, e.g., oxygen vacancies and zinc interstitials. The PL response of ZnO layer weak so circularly polarized PL can be ignored [43]. The PBG of the CNC deposited layer was precisely modulated by sonication treatment to match the ZnO bandgap in the UV region. The transmittance of the CNC/ZnO CPL photodetector decreases in general when compared with the ZnO layer and shows a significant gap between 370 and 500 nm, attributed to strong selective reflection of the photonic crystal near the PBG of the CNC layer.

CD spectra represent the difference between the absorbance of LCPL and RCPL. The CNC/ZnO photodetector shows a strong positive CD signal in the same waveband while the ZnO layer has no CD signal (Figure 4b), which indicates the left-handed chiral structure of the CNC layer. The strong positive signal with a maximum at 350 nm demonstrates that the CNC layer selectively reflects LCPL in the UV region. Since CNCs have a reported average refractive index of 1.56 [44] and chiral nematic pitch is approximately 230 nm, the PBG is measured to be 360 nm, which is in agreement with the peak of *λ* = 350 nm in CD spectra. Consequently, the chiral response region of the CNC layer is modulated to overlap the absorption edge of the ZnO layer for the high response performance of CPL photodetection in the UV region.

### 3.3. Ultraviolet Photoconductivity of ZnO Layer

**Figure 5:**
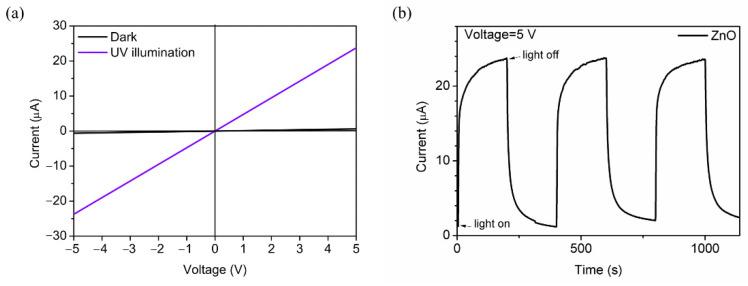
(**a**) I–V characteristics of ZnO photodetector under dark and 365 nm UV illumination. (**b**) I-t characteristics of ZnO photodetector at 5 V bias with 365 nm UV illumination switching on and off.

Figure 5a represents the I–V characteristics of ZnO layer deposited on the quartz substrate in dark or under UV light illumination. As the applied voltage increased from −5 V to 5 V, the magnitude of current change is proportional to voltage in the ZnO photodetectors annealed at 600 °C which obeys Ohm’s law, indicating the obvious ohmic contact between ZnO layer and Ag electrodes. The difference between currents in the dark and illumination represents the generation of photocurrent. The dark current is 6.07 × 10^−7^ A at the bias of 5 V. When the detector is under 365 nm UV irradiation, the light current is 2.38 × 10^−5^ A at the bias of 5 V. The light-dark current ratio is measured to 39.2, indicating the intense increase of ZnO photoconductivity under UV illumination. When the UV illumination is turned on and off, the photocurrent as a function of the time at 5 V bias is shown in Figure 5b. The rise time (defined as the time for the photocurrent to rise from 10% to 90% of the peak value) is found to be 51.3 s and the decay time (defined as the time for the photocurrent to decay from 90% to 10% of the peak value) is 118.7 s [33]. The photoconductivity of ZnO layer shows a stable behavior, which is desirable for UV detection.

The photoresponse of ZnO can be explained by the decrease and increase of conductivity due to the adsorption and desorption of oxygen molecules on the surface of the ZnO polycrystalline layer during the switch of UV illumination. In the dark condition, oxygen will be spontaneously adsorbed on the surface of ZnO where defects or traps exist. The adsorbed oxygen molecules capture free electrons and create oxygen ions which will create a thick depletion layer near the surface. Negative oxygen ions don’t participate in the free charge transport and contribute to a potential barrier, resulting in the low conductivity and dark current. When UV illumination is turned on, photogenerated electron-hole pairs are produced by the light absorption and migrate across the depletion layer, leading to the rapid decrease of photoconductivity. At the same time, the photogenerated holes recombining with oxygen ions and oxygen molecules would be desorbed from the ZnO surface, reducing the depletion layer thickness. Holes captured by oxygen ions leaves photogenerated electrons longer lifetime. The absorption and desorption of oxygen are slow processes that increase the photocurrent to the saturation value. It is critical to prepare the ZnO layer of higher crystal quality, which is expected to reduce trap density produced by defects and accelerate the recombination process of carriers [45]. 

### 3.4. CPL Detection of CNC/ZnO Photodetector

**Figure 6:**
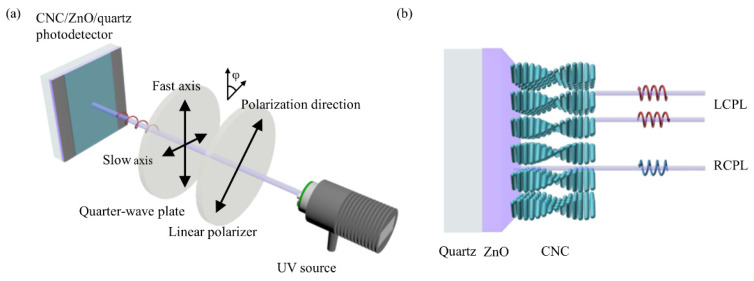
(**a**) Schematic diagram of the generation and detection of CPL. (**b**) Schematic diagram of the optical path with CNC/ZnO photodetector under the illumination of LCPL and RCPL respectively.

LCPL and RCPL are obtained through the combination of a linear polarizer and a quarter-wave plate from the same UV source at 365 nm wavelength, which have no difference except polarization states (Figure 6a). Incident lights with different polarization states travel through the CNC layer, which acts as the CPL filter that reflects back LCPL and transmits RCPL (Figure 6b). When reaching the ZnO layer, the selective reflection of CNC results in the distinction of photocurrent, where lower photocurrent is expected for LCPL and higher for RCPL.

**Figure 7:**
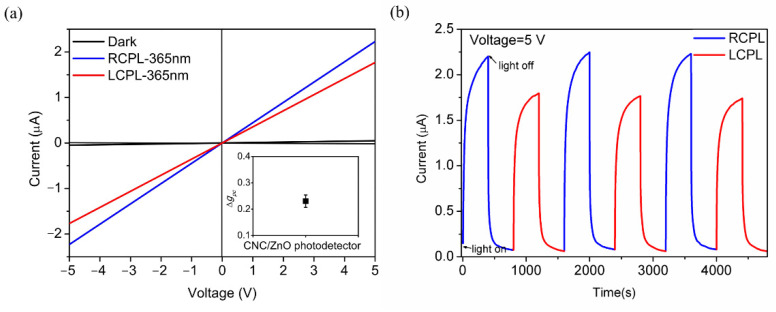
(**a**) I–V characteristics under dark, LCPL-365 nm and RCPL-365 nm illumination of CNC/ZnO photodetector. Insert: Δ*g_pc_* of CNC/ZnO photodetector. Error bar represents standard error in measurement. (**b**) I-t characteristics with recyclable response under the repetition of LCPL and RCPL switching for CNC/ZnO photodetector.

Figure 7a shows I–V characteristics under illumination of LCPL and RCPL respectively. The photocurrent is 2.23 × 10^−6^ A at 5 V under RCPL and 1.77 × 10^−6^ A at 5 V under LCPL, both showing linear relationship between photocurrent and voltage bias. The photocurrent is measured from the most stable cycles with the repetition of LCPL and RCPL from the fabricated ZnO/CNC photodetector with error bars in Figure S7. The photoconductive properties of ZnO retain in CNC/ZnO photodetector. The photocurrents under circularly polarized light decrease because of the extinction ratio of linear polarizer. There is still remarkable photocurrent discrimination between the illumination of different polarization states. The dark current of CPL detector is 4.75 × 10^−8^ A at 5 V. The photodetector has a light-dark current ratio of 46.9 and 37.2 for RCPL and LCPL, exhibiting a stable sensitivity of CPL detection. The bias of 5 V was applied and LCPL and RCPL were both illuminated for 400 s when monitoring the I-t curve of CNC/ZnO photodetector (Figure 7b). There is no attenuation in CPL detection photocurrent after the repetition of LCPL and RCPL switching in several cycles. A clear photocurrent gap exists between LCPL and RCPL illumination. The photocurrent anisotropy factor of different polarization states Δ*g_pc_* can be defined as:

(4) $$\Delta g_{pc}=\frac{2\left(I_{RCPL}-I_{LCPL}\right)}{I_{RCPL}+I_{LCPL}}.$$

Δ*g_pc_* is measured to be a competitive value of 0.23 for the strong chirality of CNC layer thanks to the macroscopic thickness (Figure S8). The rise time for LCPL and RCPL is 181.3 s and 139.7 s, which is slower than ZnO photodetector, while the decay time for LCPL and RCPL is 66.7 s and 84.3 s, which is faster than the ZnO photodetector. The mesoporous structure of CNC layer deposited on ZnO is considered to facilitate the oxygen adsorption and inhibit the desorption, resulting in the increase of rise time and the decrease of decay time [46].

**Figure 8:**
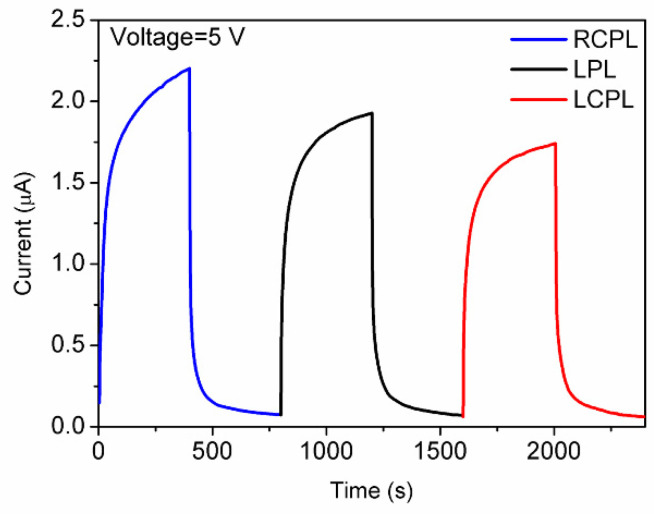
I-t characteristics with different photocurrent responses for different polarization states.

The variation in angle *φ* determines the circularity of the polarized light, changing between linear polarized light (0°) and circularly polarized light (±45°). Angle *φ* = 0° was chosen to generate linearly polarized light (LPL), which can also be regarded as a mixture of equal amounts of RCPL and LCPL. The photocurrent decreases sequentially under RCPL, LPL, and LCPL under the same bias and light intensity (Figure 8). The photocurrent is 1.93 × 10^−6^ A at 5 V under LPL. The photocurrent gap Δ*I* can be defined as:

(5) $$\Delta I=I-I_{LPL}.$$

Δ*I_RCPL_* is measured to be 3 × 10^−7^ A and Δ*I_LCPL_* is measured to be −1.6 × 10^−7^ A, showing an appealing potential to reflect the circularity of the polarized light by the anisotropy factor.

The PBG of the CNC photonic crystal has been modulated to match the semiconductor bandgap of the photosensitive ZnO layer in the UV region. CD spectra show the highest selective reflection between LCPL and RCPL near 365 nm for the PBG of the CNC photonic crystal. Figure 6b presents the schematic diagram of the optical path under the illumination of different polarization states of light, respectively. The photocurrent distinguishability originates from the preferential reflection of LCPL by the intrinsic left-handed chiral nematic structure of the CNC layer. While RCPL will mostly transmit through to reach the ZnO photosensitive layer to excite electron-hole photogeneration, which in turn increases photocurrent. The strong chirality of the CNC layer enables the circularly polarized selective photogeneration efficiency, resulting in the notable photocurrent gap between LCPL and RCPL.

## 4. Conclusions

In summary, we have successfully developed an integrated photodetector based on the combination of photoconductive ZnO and chiral photonic CNC to distinguish polarization states of CPL. ZnO shows strong absorption in the UV region. The photocurrent of 2.38 × 10^−5^ A has been achieved at the bias of 5 V with stable cycling performance. The left-handed chiral nematic structure of chiral nematic phase has been successfully retained in CNC layer by EISA. The CNC chiral photonic layer acts as a filter to reflect LCPL and transmit RCPL. The PBG was adjusted to the UV region by controlling the sonication treatment to provide a good match for the semiconductor bandgap of ZnO. The photocurrents under RCPL and LCPL are 2.23 × 10^−6^ A and 1.77 × 10^−6^ A, respectively, with the anisotropy factor Δ*g_pc_* of 0.23, and the notable photocurrent gap exists after several cycles to distinguish between different polarization states. The device realizes the high performance of polarized-sensitive detection without optical elements, offering exciting opportunities for broad application prospects in chiroptical imaging and sensing.

## Supplementary Materials

The following are available online at www.mdpi.com/article/10.3390/nano11113098/s1, Figure S1: CD spectra of CNC film cast from CNC suspension with increasing sonication treatment time of 5 min, 15 min and 20 min, Figure S2: Photograph of CNC/ZnO photodetector taken from normal direction, Figure S3: Profilometer scans of the CNC layer after EISA, Figure S4: X-ray diffraction pattern of ZnO layer, Figure S5: (**a**) Transmittance spectra of ZnO layer, (**b**) Reflectance spectra of ZnO layer, Figure S6: Room temperature PL spectra of ZnO layer, Figure S7: Photocurrent parameters of CNC/ZnO photodetectors under RCPL and LCPL, error bar represents standard error in measurement, Figure S8: (**a**) CD spectra of CNC/ZnO photodetectors with CNC suspensions of 1000 μL, 500 μL, 200 μL and 100 μL, (**b**) Normalized I-t characteristics of CNC/ZnO photodetectors with different volume of CNC suspensions.
