# Supplementary material for: Circularly Polarized Light Detection by Chiral Photonic Cellulose Nanocrystal with ZnO Photoconductive Layer in Ultraviolet Region
Source: Nanomaterials (Basel). 2021 Nov 16;11(11):3098. doi: 10.3390/nano11113098 (PMC8624577; doi:10.3390/nano11113098)
Supplement: Supplementary file 1 [file nanomaterials-11-03098-s001.zip › nanomaterials-1449799-supplementary.pdf]

nanomaterials

Supporting Information

Circularly Polarized Light Detection by Chiral Photonic Cellulose Nanocrystal with ZnO Photoconductive Layer in Ultraviolet Region

Boyu Zhang, Sixiang Zhao, Yingying Yu, Ming Li, Liancheng Zhao and Liming Gao *

State Key Laboratory of Metal Matrix Composites, School of Material Science and Engineering, Shanghai Jiao Tong University, Shanghai 200240, China; zhboyu123@sjtu.edu.cn (B.Z.); zhaosixiang@sjtu.edu.cn (S.Z.); yuyingying@sjtu.edu.cn (Y.Y.); mingli90@sjtu.edu.cn (M.L.); lczhao@sjtu.edu.cn (L.Z.)

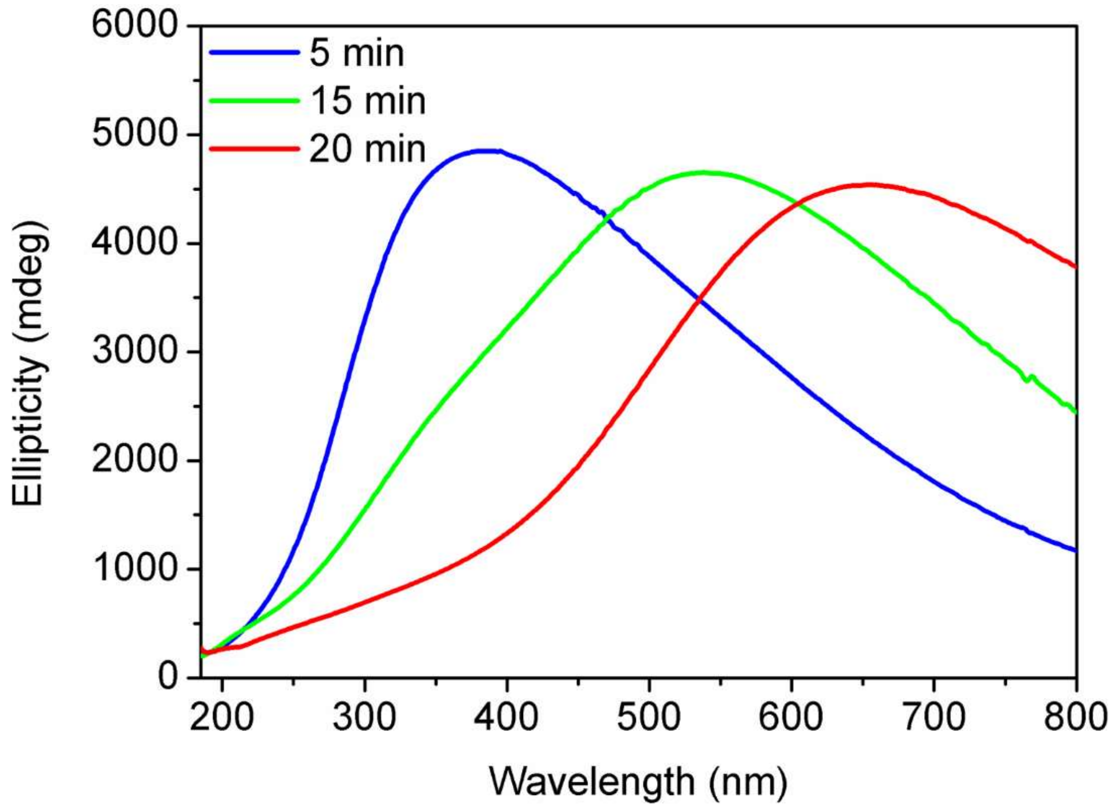

Figure S1. CD spectra of CNC film cast from CNC suspension with increasing sonication treatment time of 5 min, 15 min and 20 min

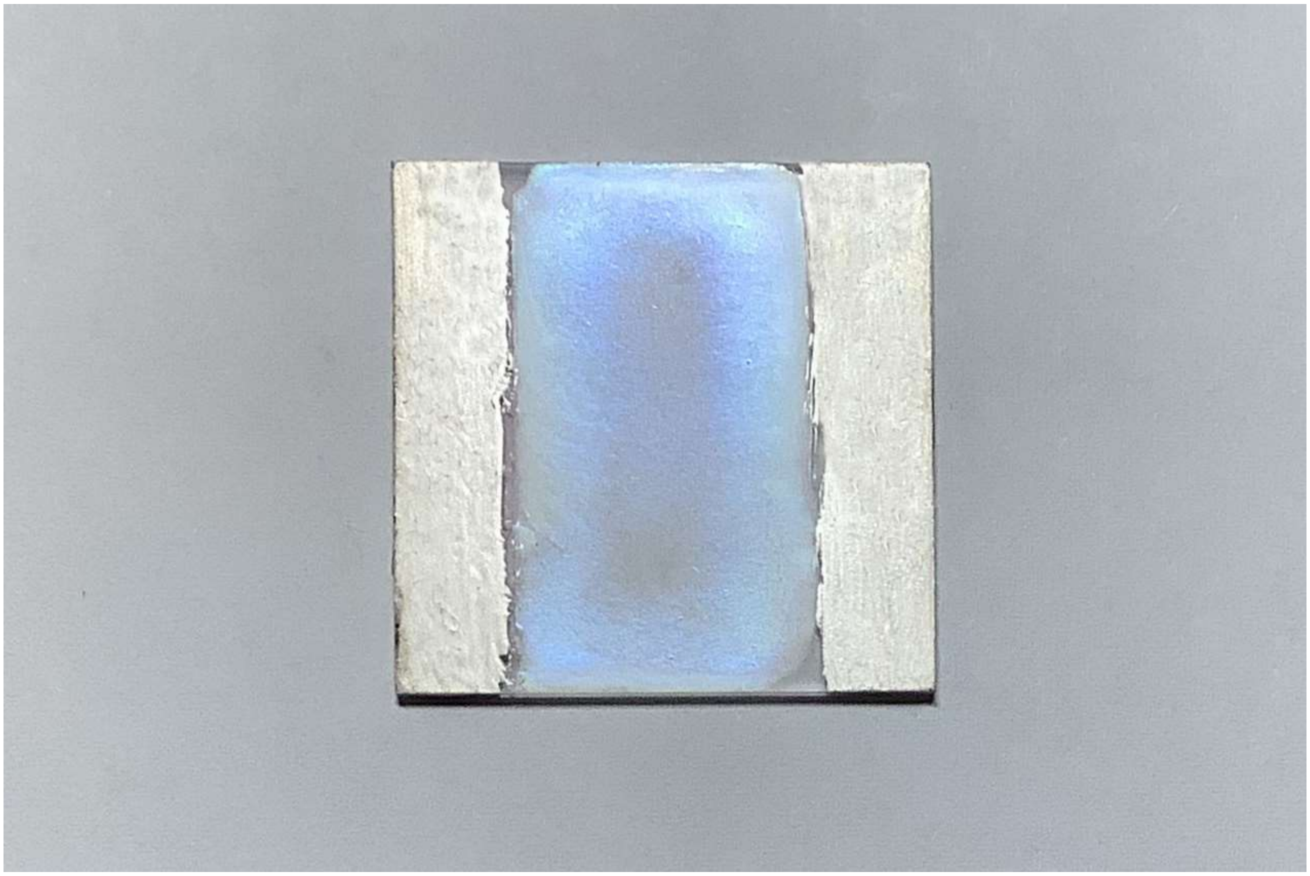

Figure S2. Photograph of CNC/ZnO photodetector taken from normal direction

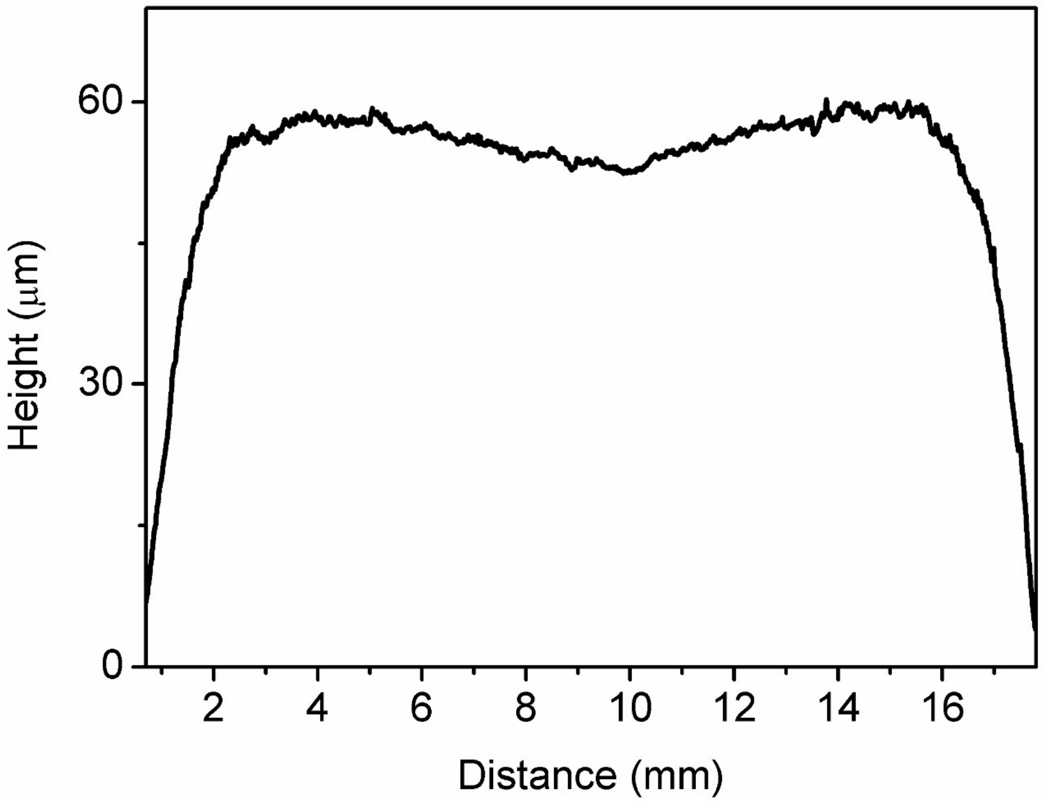

Figure S3. Profilometer scans of the CNC layer after EISA

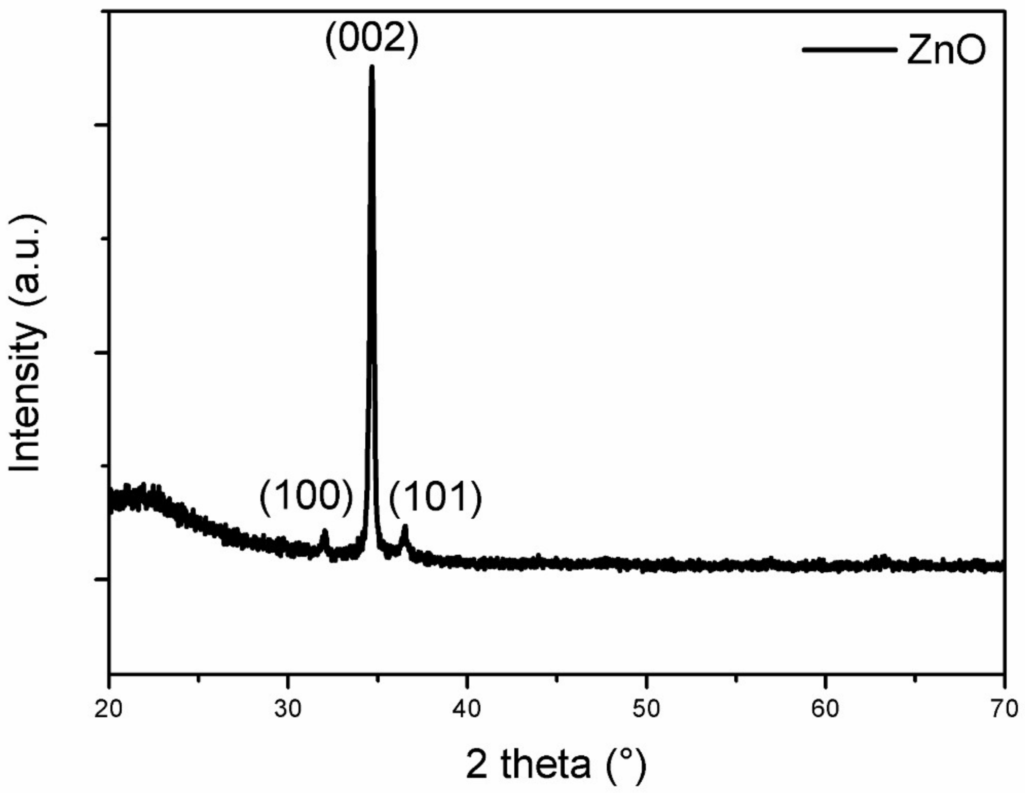

Figure S4. X-ray diffraction pattern of ZnO layer

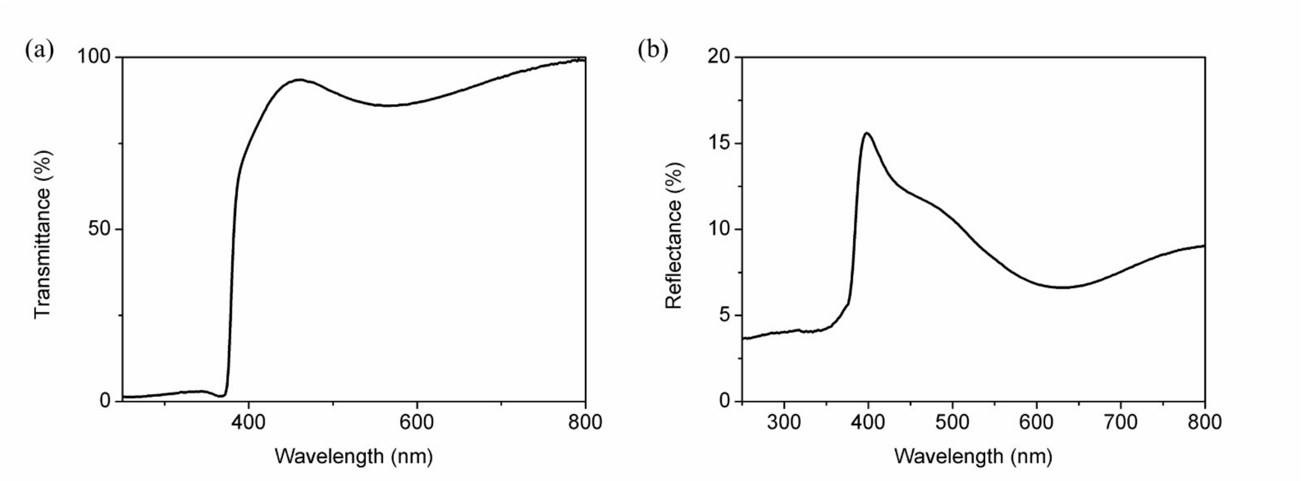

Figure S5. (**a**) Transmittance spectra of ZnO layer. (**b**) Reflectance spectra of ZnO layer.

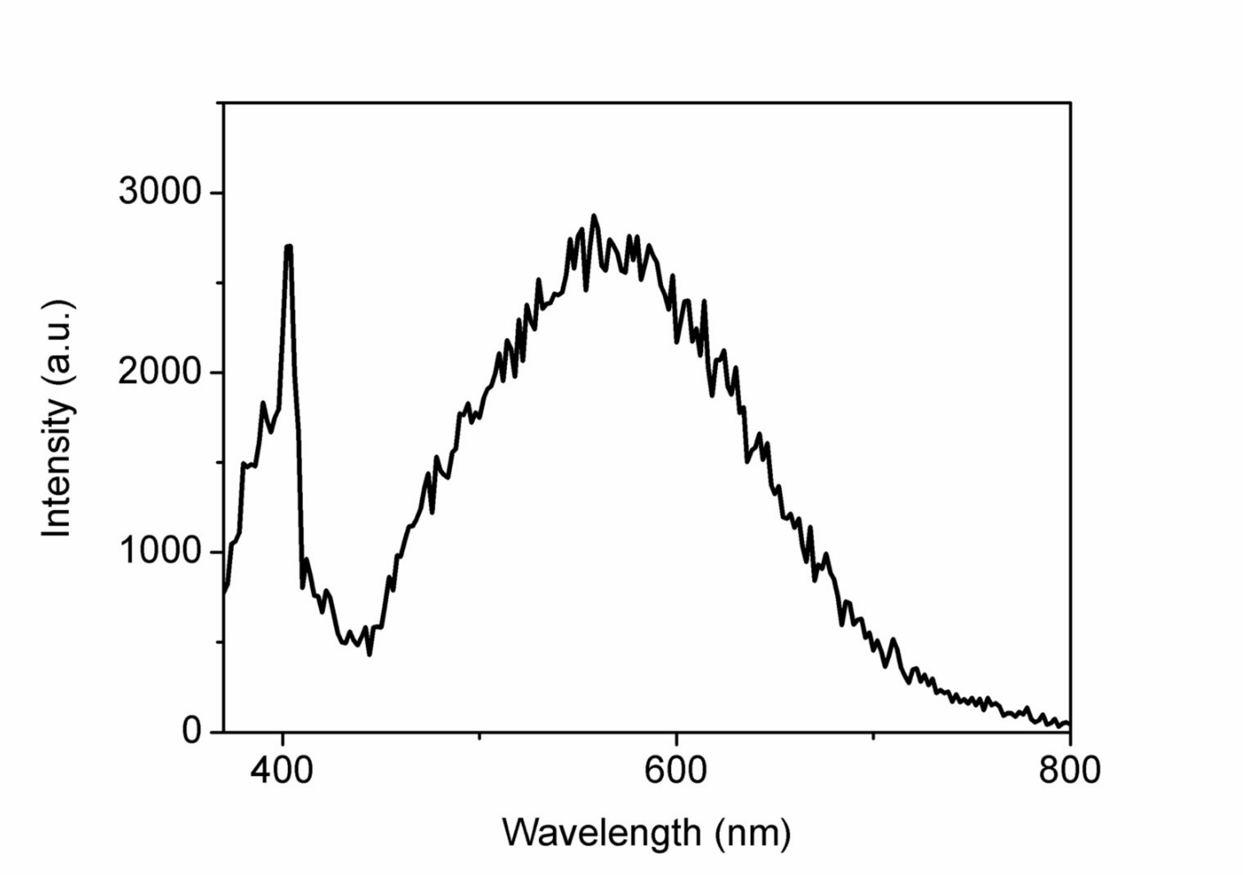

Figure S6. Room temperature PL spectra of ZnO layer

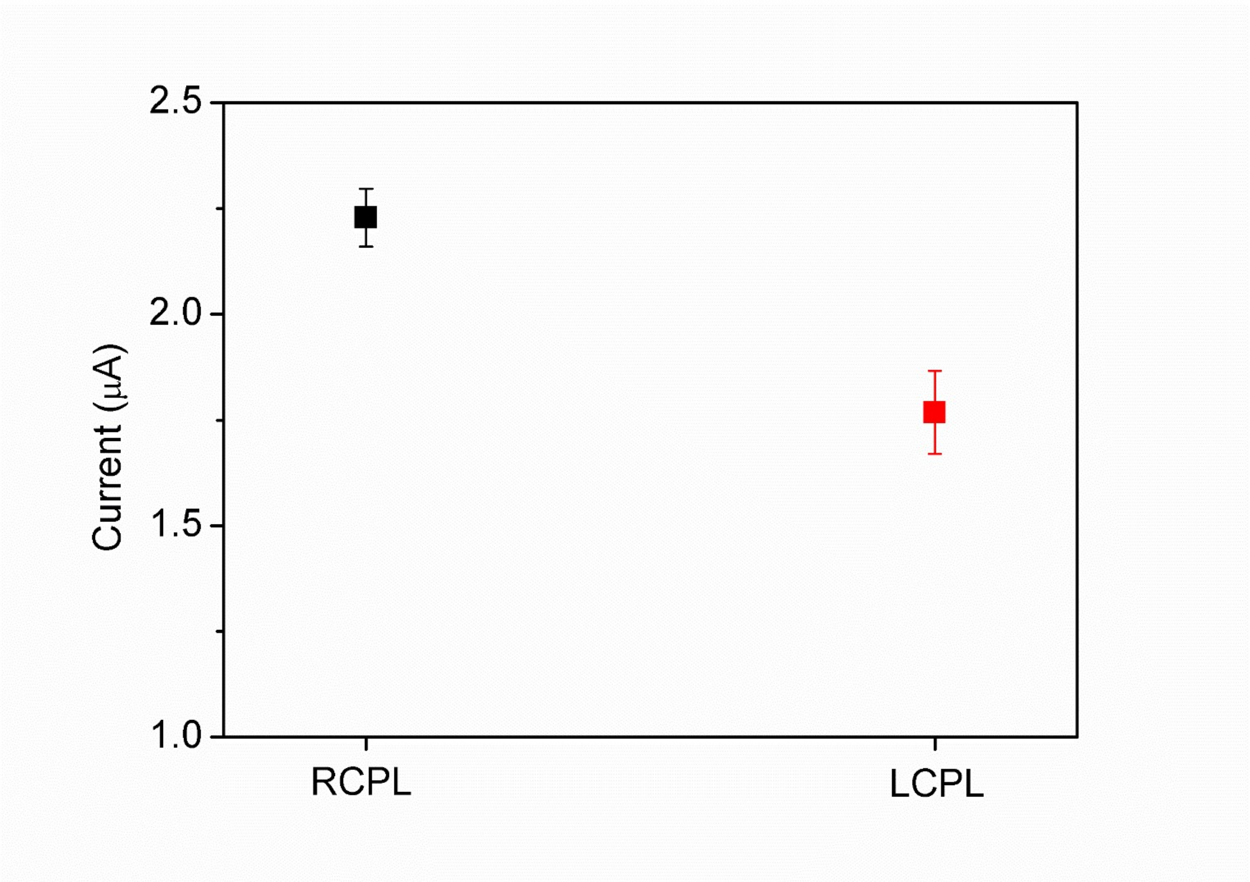

Figure S7. Photocurrent parameters of CNC/ZnO photodetectors under RCPL and LCPL. Error bar represents standard error in measurement.

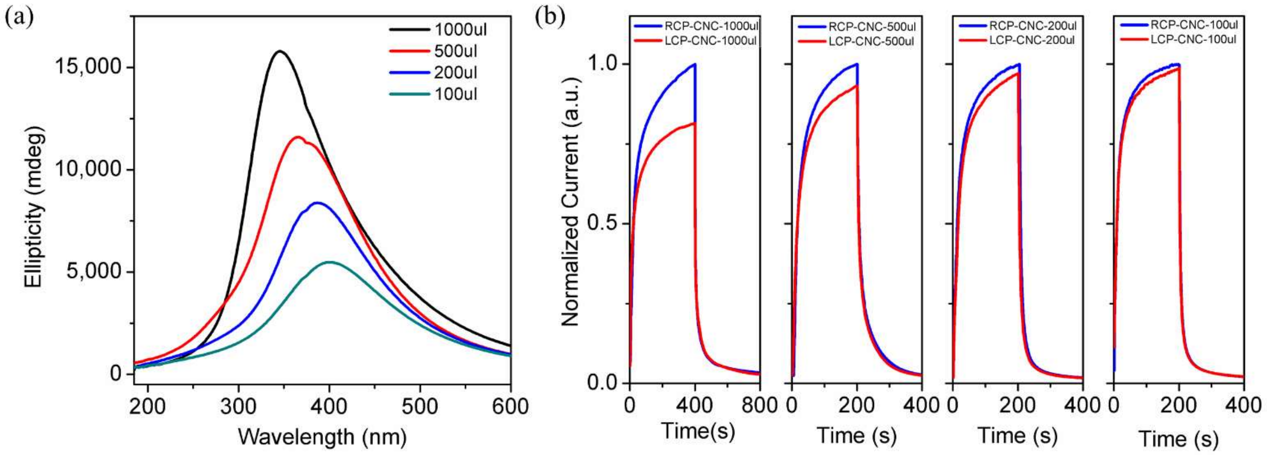

Figure S8. (**a**) CD spectra of CNC/ZnO photodetectors with CNC suspensions of 1000 μl, 500 μl, 200 μl and 100 μl. (**b**) Normalized I-t characteristics of CNC/ZnO photodetectors with different volume of CNC suspensions.
